# Rational screening of peroxisome proliferator-activated receptor-γ agonists from natural products: potential therapeutics for heart failure

**DOI:** 10.1080/13880209.2016.1255648

**Published:** 2016-12-09

**Authors:** Rui Chen, Jing Wan, Jing Song, Yan Qian, Yong Liu, Shuiming Gu

**Affiliations:** a Department of Geriatric Medicine, Shanghai Eighth People's Hospital, Shanghai, China;; b Department of Cardiology, Shanghai Eighth People's Hospital, Shanghai, China

**Keywords:** Natural medicine discovery, virtual screening, brain natriuretie peptide, protein-ligand recognition

## Abstract

**Context:** Peroxisome proliferator-activated receptor-γ (PPARγ) is a member of the nuclear hormone receptor superfamily of ligand-activated transcription factors. Activation of PPARγ pathway has been shown to enhance fatty acid oxidation, improve endothelial cell function, and decrease myocardial fibrosis in heart failure. Thus, the protein has been raised as an attractive target for heart failure therapy.

**Objective:** This work attempted to discover new and potent PPARγ agonists from natural products using a synthetic strategy of computer virtual screening and transactivation reporter assay.

**Materials and methods:** A large library of structurally diverse, drug-like natural products was compiled, from which those with unsatisfactory pharmacokinetic profile and/or structurally redundant compounds were excluded. The binding mode of remaining candidates to PPARγ ligand-binding domain (LBD) was computationally modelled using molecular docking and their relative binding potency was ranked by an empirical scoring scheme. Consequently, eight commercially available hits with top scores were selected and their biological activity was determined using a cell-based reporter-gene assay.

**Results:** Four natural product compounds, namely ZINC13408172, ZINC4292805, ZINC44179 and ZINC901461, were identified to have high or moderate agonistic potency against human PPARγ with EC_50_ values of 0.084, 2.1, 0.35 and 5.6 μM, respectively, which are comparable to or even better than that of the approved PPARγ full agonists pioglitazone (EC_50 _=_ _0.16 μM) and rosiglitazone (EC_50 _=_ _0.034 μM). Hydrophobic interactions and van der Waals contacts are the primary chemical forces to stabilize the complex architecture of PPARγ LBD domain with these agonist ligands, while few hydrogen bonds, salt bridges and/or π-π stacking at the complex interfaces confer selectivity and specificity for the domain-agonist recognition.

**Discussion and conclusion:** The integrated *in vitro*-*in silico* screening strategy can be successfully applied to rational discovery of biologically active compounds. The newly identified natural products with PPARγ agonistic potency are considered as promising lead scaffolds to develop novel chemical therapeutics for heart failure.

## Introduction

Peroxisome proliferator-activated receptors (PPARs) belong to superfamily of phylogenetically related protein termed nuclear hormone factor, which comprise of three subtypes: PPARα, PPARγ and PPARβ/δ (Tyagi et al. 2011). The PPARγ regulates various neurohumoral factors involved in the progression of heart failure, a disease marked by abnormal myocardial metabolism, fibrosis and insulin insensitivity. Its activating ligands (agonists) inhibit cardiac hypertrophy and ischaemia-reperfusion injury *via* a PPARγ-independent pathway (Ehara et al. 2004). Several clinical and preclinical studies have demonstrated the beneficial effects of PPARγ agonists on various cardiovascular risk factors (Das & Chakrabarti 2006). Wojtkowska et al. (2014) observed that PPARγ expression level is considerably upregulated during development of heart failure in patients with coronary artery disease after coronary artery bypass-grafting. Accumulated evidence suggested that the production of tumour necrosis factor α (TNFα) by cardiac myocytes promotes the development and progression of heart failure, and PPARγ agonists can potently inhibit the cardiac expression of TNFα through attenuating NF-κB activation, suggesting that treatment with the agonists may prevent the development of congestive heart failure (Takano et al. 2000). In addition, PPARγ has also been established as a sophisticated target of diabetes mellitus, one of the leading and growing causes of coronary artery disease and heart failure (Kasznicki & Drzewoski 2014).

PPARγ agonists represent a heterogeneous group of compounds that have been used in the treatment of cardiovascular and metabolic diseases for decades. While the primary indications for PPARγ agonist therapy focus on hyperlipidemia and diabetes, there is a growing body of preclinical data that suggests they may be beneficial in the treatment of heart failure. PPARγ agonist treatment in numerous animal models of systolic heart failure have demonstrated improvement in cardiac function with decreased fibrosis, improved contractility and endothelial function (Sarma 2012). In the present study, we attempted to discover new and potent PPARγ agonists from natural product compounds. Natural products have proven historically to be a promising pool of structures for drug discovery, and a significant research effort has recently been undertaken to explore the PPARγ-activating potency of a wide range of natural products originating from traditionally used medicinal plants or dietary sources (Wang et al. 2014). However, their use has diminished in the past two decades, in part because of technical barriers to screening natural products in high-throughput manner against molecular targets (Harvey et al. 2015). Here, a computational protocol that integrates pharmacokinetics analysis, chemical redundancy reduction, flexible molecular docking and binding affinity prediction is described. The protocol was used to perform high-throughput virtual screening against a distinct library of structurally diverse, drug-like natural products. The biological activity of few identified compounds promising as PPARγ agonists was determined using cell-based reporter-gene assays. The structural basis and energetic property of potent agonists binding to the ligand-binding domain (LBD) of PPARγ were also investigated in detail to elucidate the molecular mechanism and biological implication underlying PPARγ–agonist recognition and association.

## Materials and methods

### Pharmacokinetics analysis

The pharmacokinetic profile of natural product compounds, namely ADMET (absorption, distribution, metabolism, excretion and toxicity), was predicted using PreADMET method (Lee et al. 2004). The PreADMET describes the ADMET properties of a potential drug candidate by reproducing *in vitro* or *in vivo* assay models, including human intestinal absorption (HIA), Caco-2 cell permeability (Caco-2), Maden Darby Canine Kidney (MDCK) cell permeability, blood–brain barrier (BBB) penetration, mutagenicity and carcinogenicity. Here, we employed a set of ADMET criteria proposed by Gong et al. (2016) to filter compound candidates with unwanted ADMET properties (Table 1).

**Table 1.** The ADMET criteria (Gong et al. 2016).

ADMET	Type	Criterion
Caco-2	Absorption	>4 nm/s
HIA	Absorption	>20%
MDCK	Absorption	>25 nm/s
BBB	Distribution	>0
Mutagenicity	Toxicity	Negative
Carcinogenicity	Toxicity	Negative

### Chemical redundancy

The chemical redundancy of a natural product library can be characterized in a pairwise manner using the structural similarity between all pairs of compounds in the library. The characterization was based on 1024-bit hashed Morgan fingerprint (Yu et al. 2015), which is a circular fingerprint implemented in the open-source cheminformatics software RDKit (http://rdkit.org). The Tanimoto coefficient (TC) was chosen to measure chemical similarity between the fingerprints of two compounds (Nikolova & Jaworska 2003). The TC similarity with a binary fingerprint is defined as:

(1) $$\mathrm{TC}=\frac{c}{a+b-c}$$

where *a* and *b* are the number of fingerprint bits of two compared compounds, and *c* is the common bits shared by the two compounds. In this study, if any compound pair has a high structural similarity with TC >0.85, one of them would be removed from the library to reduce chemical redundancy. The cutoff value TC >0.85 has been widely used in the cheminformatics community to characterize compounds with similar structural property and biological activity (Chen et al. 2015).

### Molecular docking and virtual screening

Currently, more than 100 of PPARγ crystal structures are decomposed in the RCSB protein data bank (PDB) database (Berman et al. 2000), of which most were in complex with a ligand. According to the suggestion of Lewis et al. (2015), structures without cocrystallized ligands, with large groups of missing residues, with multiple side chain positions in binding site residues, with covalently bound ligands, with cocrystallized ligands that fit other ligand categories, with multiple distinct locations for ligand binding, and with ambiguous cocrystallized ligand-binding patterns were excluded because these characteristics would introduce less reliable or less appropriate atom coordinate data. Consequently, the complex crystal structure of PPARγ LBD domain with its full agonist rosiglitazone (PDB: 4ema) was selected as template to perform molecular docking and high-throughput virtual screening. All water molecules, ions and cofactors were removed manually from the structure. A set of spheres representing ligand-binding cavity in the domain was defined in terms of the cocrystallized rosiglitazone, and atomic types, partial charges and protonation state of the cavity residues were assigned using UCSF Chimera (Pettersen et al. 2004).

Molecular docking calculations were implemented with UCSF DOCK3.6 program (Irwin et al. 2009). The docking was performed against the prepared structure of PPARγ LBD domain using a strategy described by Heusser et al. (2013). The flexible ligand-sampling algorithm in DOCK superimposes a docked molecule onto the matching spheres. The spheres were also labelled for chemical matching based on the local receptor environment (Shoichet & Kuntz 1993). The degree of ligand sampling is determined by the bin size, bin size overlap and distance tolerance. For ligand conformations passing an initial steric filter, a physics-based scoring function was used to evaluate the fit to the binding site. For the best-scoring conformation of each docked molecule, 100 steps of rigid-body minimization were carried out. The score for each conformation was calculated as the sum of electrostatic and van der Waals interaction energies between the protein receptor and docked molecule, corrected for ligand desolvation (Shoichet et al. 1999). All natural product candidates were docked and ranked based on their predicted binding scores.

### Transactivation reporter assay

The agonistic potency (EC_50_) of tested compounds against human PPARγ (hPPARγ) was measured as transactivation activity using a standard reporter-gene assay protocol described previously (Berger et al. 1999; Kasai et al. 2008). Briefly, chimeric receptor expression plasmid pcDNA3-hPPARγ/GAL4 was prepared by inserting the yeast GAL4 transcription factor DNA-binding domain adjacent to the hPPARγ LBD domain. The reporter plasmid, p(UAS)_5_-*tk*-*luc*, was generated by inserting five copies of the GAL4 response element upstream of the herpesvirus minimal thymidine kinase promoter and the luciferase reporter gene. The chimeric receptor expression plasmid and reporter plasmid were cotransfected into CV-1 cells. The transfected cells were cultured with or without each tested compound for 48 h and then steady-state luminescence intensity was measured with a microplate luminometer (Turner Biosystems). All compounds were purchased from Sigma-Aldrich and ChemPartner, and dissolved in dimethyl sulfoxide (<0.1% v/v). Each assay was carried out in triplicate.

## Results and discussion

### Docking validation

**Figure 1.**
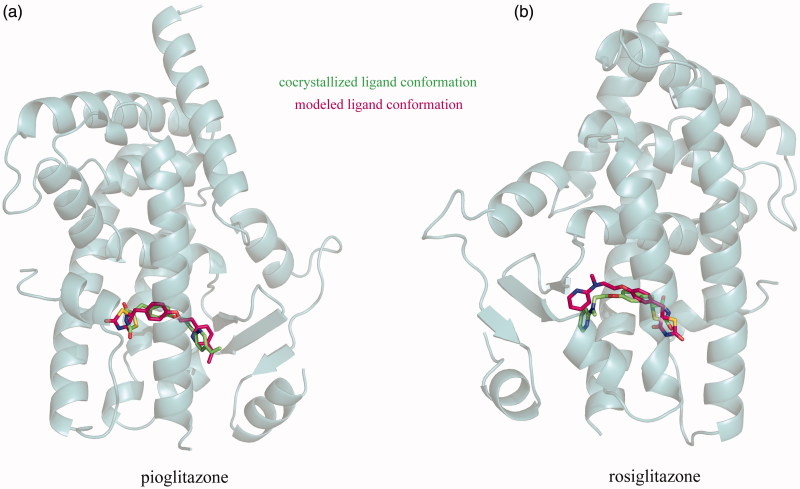
Structure superposition of docking-modelled PPARγ LBD domain complexes with pioglitazone (a) and rosiglitazone (b) onto their crystal counterparts (PDB: 2xkw and 4ema, respectively), resulting *rmsd* values of 0.35 and 0.72 Å, respectively. The agonist ligands and domain are shown in stick and ribbon styles, respectively.

Two FDA-approved PPARγ agonists, i.e., pioglitazone and rosiglitazone, were used to test the capability of UCSF DOCK3.6 molecular docking (Irwin et al. 2009) to reconstruct the agonist complex structures with PPARγ LBD domain. The docking calculations were performed to separately model the binding modes of the two agonists to the domain in a blind manner, which were then compared to the high-resolution crystal structures of domain cocrystallized with pioglitazone (PDB: 2xkw) and rosiglitazone (PDB: 4ema) retrieved from the PDB database (Berman et al. 2000). Here, the computationally modelled complexes were superposed onto crystal structures using SPDBV4.1 software (Guex & Peitsch 1997). As can be seen in Figure 1, the docking calculations can well reproduce the direction and location of pioglitazone and rosiglitazone in the ligand-binding cavity of PPARγ LBD domain, resulting in root-mean-square deviation (*rmsd*) values of 0.35 and 0.72 Å between the crystal and modelled conformations of the two agonist ligands, respectively. This is satisfactory if considering that the pioglitazone and rosiglitazone are highly flexible with a linear structure containing a number of rotatable dihedral angles that are difficult to be computationally modelled reliably for molecular docking methodology.

**Figure 2.**
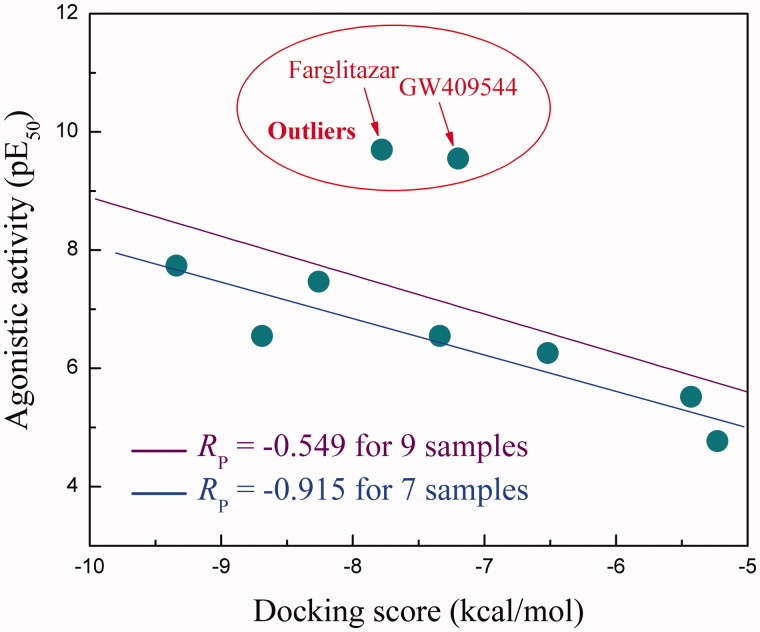
Scatter plot of agonistic activity against docking score over nine PPARγ agonists.

Next, we tested the statistical correlation between the docking score and biological activity of agonist ligands against PPARγ. A total of nine PPARγ agonists with experimentally agonistic activity (EC_50_ value) were collected from a variety of literatures (Table 2) (Willson et al. 1996, 2000; Xu et al. 2001; Dey et al. 2003; Schupp et al. 2006; Kasai et al. 2008), in which three (pioglitazone, rosiglitazone and troglitazone) have been approved and others (farglitazar, GW409544, ciglitazone, E3030, CLX-0921 and EXP3179) are in clinical or preclinical development. These compounds are diverse in terms of their structures (polar, charged, aromatic and hydrophobic) and biological activities (EC_50_ values range between 0.20 and 17000 nM). Therefore, they represent a heterogeneous set of PPARγ response to diverse agonists. The complex structures of PPARγ LBD domain with these compounds were computationally modelled *via* molecular docking, and their binding strengths were predicted using docking scores based on the best modelled conformation of agonist ligands. The obtained scores were plotted against experimental activities in Figure 2 and tabulated in Table 2 for comparison purpose. As might be expected, a moderate linear correlation between the calculated scores and experimental activities can be observed with Pearson’s correlation coefficient *R*_p_ = −0.549. By visually examining the plot, it is evident that two outliers can be found, namely farglitazar and GW409544. The two compounds have a bulky structure and exhibit highly flexible scaffolds that may not be described reliably by molecular docking calculations. Thus, their agonistic activities seem to be underestimated considerably by docking scores. Here, we just removed the two compounds and re-analyzed score-activity correlation for the remaining seven samples. As might be expected, the Pearson’s coefficient *R*_p_ increases largely from −0.549 to −0.915 and no obvious outliers can be found, indicating a significant statistical correlation (Figure 2).

**Table 2.** The nine PPARγ agonists with known agonistic activity.

			Agonistic activity	
Agonist	Molecular structure	Docking score (kcal/mol)	EC_50_ (nM)	pEC_50_
Pioglitazone		−8.69	280 (Xu et al. 2001)	6.55
Rosiglitazone		−9.34	18 (Xu et al. 2001)	7.74
Farglitazar		−7.78	0.20 (Xu et al. 2001)	9.70
GW409544		−7.20	0.28 (Xu et al. 2001)	9.55
Troglitazone		−6.52	550 (Willson et al. 2000)	6.26
Ciglitazone		−5.43	3000 (Willson et al. 1996)	5.52
E3030		−8.26	34 (Kasai et al. 2008)	7.47
CLX-0921		−7.34	284 (Dey et al. 2003)	6.55
EXP3179		−5.23	17,000 (Schupp et al. 2006)	4.77

### Virtual screening and activity assay

A total of ∼190,000 natural products, metabolites and biogenic compounds were retrieved from the ZINC database (Irwin & Shoichet 2005). These compounds are lead-like and commercially available, and their molecular structures were pre-deposited in the database in a readily compatible format with high-throughput virtual screening. First, the pharmacokinetic profile of compound candidates was analyzed using PreADMET method (Lee et al. 2004), which predicted six ADMET properties for a compound, including HIA, Caco-2, MDCK cell permeability, BBB penetration, mutagenicity and carcinogenicity. Here, we employed the criteria listed in Table 1 to exclude those candidates with unwanted properties. Subsequently, the structural diversity of compounds was characterized by Morgan fingerprint and measured using TC similarity coefficient; compounds with TC >0.85 were removed from library to reduce chemical redundancy. Consequently, ∼97,000 structurally diverse compounds with satisfactory pharmacokinetic profile were remained, which were one-by-one docked into the active cavity of PPARγ LBD domain automatically using UCSF DOCK3.6 program (Irwin et al. 2009) based on a flexible ligand-sampling algorithm. The docking-based high-throughput virtual screening is an exhaustive process but can rank the relative binding strength of compound candidates to the domain in a statistically significant manner.

**Figure 3.**
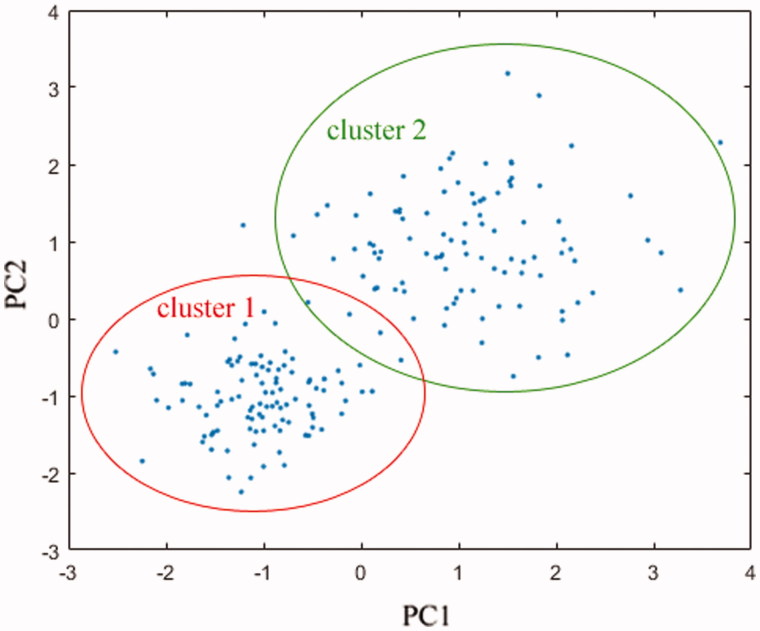
PCA mapping of docking-predicted top-100 domain binders onto the first two principal component spaces PC1 and PC2.

Here, the top-100 domain binders ranked by docking score were examined manually, and they were mapped onto a 2D-plot using principal component analysis (PCA) (Jolliffe & Cadima 2016) based on structural fingerprints. As can be seen in Figure 3, these sample points are distributed unevenly across the first two principal component spaces, which can roughly be clustered into two regions, the clusters 1 and 2, with similarity coefficient less than 0.5. The cluster 1 comprises 43 samples, many of which have a common scaffold of stringing a number of aromatic rings and/or heterocycles, while the cluster 2 contains 55 samples that exhibit a wider structural diversity. The clusters may represent two major groups of PPARγ ligands that can potently bind to LBD domain in different conformations. Here, eight commercially available compounds, three from cluster 1, four from cluster 2 and one outlier, were selected to evaluate their agonistic activity against human PPARγ using cell-based transactivation reporter assay (Table 2). To validate our chimeric GAL4-PPAR transactivation reporter assay, we measured the PPARγ transactivation activity of two approved agonists in our assay. The EC_50_ values of pioglitazone and rosiglitazone for PPARγ were determined to 0.16 and 0.034 μM, respectively; the values were roughly close to those of a previous report (0.28 and 0.018 μM, respectively (Xu et al. 2001)), suggesting that our assay is reliably and can be applied for the eight natural product hits.

Four out of the eight tested compounds were found to have a high or moderate agonistic activity with EC_50_ values ranging between 0.084 and 5.6 μM (Table 3). In particular, the compound ZINC13408172 was determined to have a strong potency at nanomolar level (EC_50 _=_ _0.084 μM), which is comparable to or better than that of the approved agents pioglitazone (EC_50 _=_ _0.16 μM) and rosiglitazone (EC_50 _=_ _0.034 μM). The ZINC13408172 is a flexible molecule that can be well compatible with the allosteric cleft of PPARγ LBD domain to shape a good physicochemical complementarity between them. In addition, other three compounds ZINC4292805, ZINC44179 and ZINC901461 also exhibited activating capability against PPARγ with EC_50_ values of 2.1, 0.35 and 5.6 μM, respectively. However, no agonistic activity of the tested natural products ZINC3846920, ZINC3848501, ZINC6096559 and ZINC1595957 was observed, suggesting that there is a bias between theoretical prediction and experimental assay. This is acceptable if considering the high hit rate (50%) achieved in this study.

**Table 3.** The eight identified compounds as well as two approved PPARγ agonists.

ZINC	Molecular structure	Docking score (kcal/mol)	EC_50_ (μM)	Agonistic intensity^c^
3846920		−9.18	n.d.	n.d.
4292805		−10.34	2.1 ± 0.29	63%
44179		−9.87	0.35 ± 0.06	78%
3848501		−9.20	n.d.	n.d.
13408172		−9.54	0.084 ± 0.015	116%
901461		−9.12	5.6 ± 0.72	37%
6096559		−9.43	n.d.	n.d.
1595957		−9.86	n.d.	n.d.
Pioglitazone^a^		−8.69	0.16 ± 0.023 (0.28^b^)	100%
Rosiglitazone^a^		−9.34	0.034 ± 0.006 (0.018^b^)	124%

a Two existing PPARγ agonists for comparison purpose.

b Reported in Ref. (Xu et al. 2001).

c The percentage of agonistic potency relative to full agonist Pioglitazone at their respective EC_50_ concentration.

### Structure-based analysis of agonist interaction with PPARγ LBD domain

**Figure 4.**
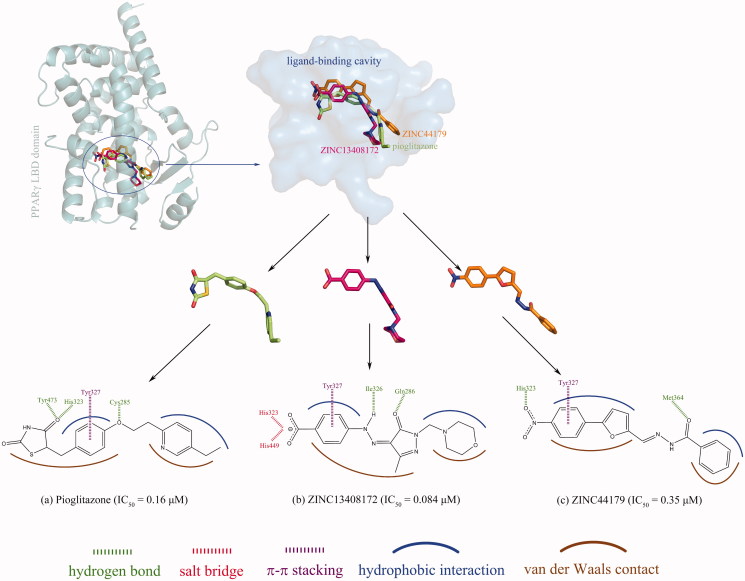
Superposition of computationally modelled conformations of ZINC13408172 and ZINC44179 onto that of pioglitazone in the ligand-binding cavity of PPARγ LBD domain. Comparison of noncovalent interactions at the complex interfaces of PPARγ LBD domain with pioglitazone (a), ZINC13408172 (b) and ZINC44179 (c).

Here, the computationally modelled conformations of two newly identified potent PPARγ agonists, i.e., ZINC13408172 and ZINC44179 (EC_50 _=_ _0.084 and 0.35 μM, respectively), were superposed onto the cocrystallized structure of pioglitazone in the ligand-binding pocket of PPARγ LBD domain (Figure 4). It is evident that the ZINC13408172 and ZINC44179 have a similar binding mode with the approved agent pioglitazone; all of them exhibit a ‘C’ shape that were also be observed in the binding of other existing agonists such as rosiglitazone and troglitazone to PPARγ, suggesting that the potent PPARγ agonists should share a flexible linear structure with a chain of aromatic rings and/or heterocycles. The noncovalent interactions at the complex interfaces of PPARγ LBD domain with pioglitazone, ZINC13408172 and ZINC44179 are also compared in Figure 4. As might be expected, the three agonists form similar interaction patterns with the domain, where the hydrophobic interactions and van der Waals contacts are the primary chemical forces to stabilize the complex architecture of PPARγ LBD domain with agonist ligands, while few hydrogen bonds, salt bridges and/or π–π stacking at the complex interfaces confer selectivity and specificity for the domain–agonist recognition.
